# Carry-over effects of urban larval environments on the transmission potential of dengue-2 virus

**DOI:** 10.1186/s13071-018-3013-3

**Published:** 2018-07-17

**Authors:** Michelle V. Evans, Justine C. Shiau, Nicole Solano, Melinda A. Brindley, John M. Drake, Courtney C. Murdock

**Affiliations:** 1 Odum School of Ecology, University of Georgia, Athens, GA USA; 2 Center for the Ecology of Infectious Diseases, University of Georgia, Athens, GA USA; 3 Department of Infectious Disease, University of Georgia, Athens, GA USA; 4 Department of Population Health, University of Georgia, Athens, GA USA; 5 Center for Tropical Emerging Global Diseases, University of Georgia, Athens, GA USA; 6 Center for Vaccines and Immunology, University of Georgia, Athens, GA USA; 7 River Basin Center, University of Georgia, Athens, GA USA

**Keywords:** *Aedes albopictus*, Dengue, Carry-over effects, Urban microclimate

## Abstract

**Background:**

Mosquitoes are strongly influenced by environmental temperatures, both directly and indirectly *via* carry-over effects, a phenomenon by which adult phenotypes are shaped indirectly by the environmental conditions experienced in previous life stages. In landscapes with spatially varying microclimates, such as a city, the effects of environmental temperature can therefore lead to spatial patterns in disease dynamics. To explore the contribution of carry-over effects on the transmission of dengue-2 virus (DENV-2), we conducted a semi-field experiment comparing the demographic and transmission rates of *Aedes albopictus* reared on different urban land classes in the summer and autumn season. We parameterized a model of vectorial capacity using field- and literature-derived measurements to estimate the bias introduced into predictions of vectorial capacity not accounting for carry-over effects.

**Results:**

The larval environment of different land classes and seasons significantly impacted mosquito life history traits. Larval development and survival rates were higher in the summer than the autumn, with no difference across land class. The effect of land class on adult body size differed across season, with suburban mosquitoes having the smallest wing length in the summer and the largest wing length in the autumn, when compared to other land classes. Infection and dissemination rates were higher in the autumn and on suburban and rural land classes compared to urban. Infectiousness did not differ across land class or season. We estimate that not accounting for carry-over effects can underestimate disease transmission potential in suburban and urban sites in the summer by up to 25%.

**Conclusions:**

Our findings demonstrate the potential of the larval environment to differentially impact stages of DENV-2 infection in *Ae. albopictus* mosquitoes *via* carry-over effects. Failure to account for carry-over effects of the larval environment in mechanistic models can lead to biased estimates of disease transmission potential at fine-scales in urban environments.

**Electronic supplementary material:**

The online version of this article (10.1186/s13071-018-3013-3) contains supplementary material, which is available to authorized users.

## Background

Climate plays an important role in the transmission of mosquito-borne pathogens, determining the geographic range of disease vectors and shaping transmission dynamics [1, 2]. Heterogeneity in environmental conditions can directly shape individual-level variation in traits relevant to mosquito population dynamics [3] and pathogen transmission [4]. In addition to these direct effects, mosquito phenotypes can be shaped indirectly by the environmental conditions experienced in previous life history stages, a phenomenon known as carry-over effects [5]. Carry-over effects have been documented in a wide-range of species with complex life-cycles, such as amphibians [6], migratory birds [7], and damselflies [8]. Similarly, the mosquito life-cycle is characterized by ontogenetic niche shifts, with a larval aquatic stage and an adult terrestrial stage. Following these studies, we reason that the thermal environment a mosquito experiences during its larval stage is likely to have lasting impacts on adult traits, and, ultimately, on transmission potential.

Although it has been previously demonstrated that larval environmental temperature can alter individual mosquito traits important for transmission [9, 10], the net effect of temperature-mediated carry-over effects on overall transmission potential is ambiguous. Current models of mosquito-borne disease typically only incorporate direct effects of temperature, despite evidence that carry-over effects can have large impacts on adult phenotypes [11–13]. Additionally, laboratory studies designed to estimate temperature-mediated carry-over effects are often conducted across a wider range of temperatures than mosquitoes typically experience in the field [14]. The studies are not easily “scaled-up” to explain transmission across a landscape when incorporated into temperature-dependent models of mosquito-borne disease [15]. Urban landscapes, in particular, are composed of a variety of microclimates, which can differentially impact mosquito life-history traits leading to heterogeneity in vector population dynamics across the landscape [16]. However, it is unknown if variation in microclimate across an urban area also has implications for carry-over effects of the larval environment on adult phenotypes.

We hypothesize that relevant environmental variation across an urban landscape during the larval stage will have lasting impacts on adult traits that are important for mosquito population dynamics and pathogen transmission. Further, we predict that failure to account for carry-over effects will result in a biased estimate of vectorial capacity, the rate at which future infections arise from one infectious mosquito. To estimate the effects of the larval environment in a spatially heterogeneous, urban environment, we conducted a semi-field experiment exploring population and dengue-2 virus (DENV-2) transmission relevant life-history traits from *Aedes albopictus* mosquitoes reared in three urban land classes across the summer and autumn. We used a mixture of field-derived and temperature-dependent parameters to construct a model of vectorial capacity. Our modeled vectorial capacity was then compared to a calculation using the experimental grand mean for parameters affected by carry-over effects in order to estimate the bias introduced by not including these indirect effects.

## Methods

**Fig. 1:**
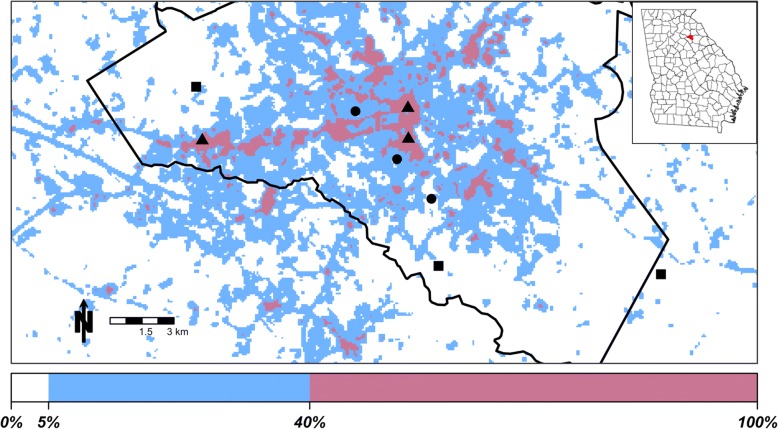
Map of study sites in Athens, GA, USA. Inset illustrates location of Athens-Clarke County (black outline) in the state of Georgia. Symbols represent land classes (square: rural; circle: suburban; triangle: urban). Colors represent the amount of impervious surface within the 210 m focal area of each pixel, as illustrated on the color bar on the bottom

We conducted a semi-field experiment across an urban gradient in Athens, GA, USA, in the summer and autumn of 2016. To explore the effects of microclimate variation across an urban landscape, we used an impervious surface map (National Land Cover Database 2011 [17]) to select three replicate sites 30 × 30 m each of low (0–5%), intermediate (6–40%), and high (41–100%) impervious surface. Percent impervious surface is an accurate predictor of land surface temperature, particularly for urban landscapes [18], and allowed us to ensure our sites exhibited the full range of urban microclimates. To select our sites, we calculated the percent impervious surface of each 30 × 30 m pixel using a moving focal window of 210 × 210 m, as the surrounding impervious surface can affect the microclimate in the pixel of interest. We then classified each pixel based on the mean impervious surface within its focal window, with 0–5% representing low, 6–40% representing intermediate, and 41–100% representing high. Because impervious surface is an effective classifier of urban land classes [19], we identified the sites as rural, suburban, and urban with low, intermediate, and high impervious surface scores, respectively. Final site selection was constrained by access and permissions, however, the final distribution of sites was chosen to ensure all sites were at least 3 km from others of the same land class, and were interspersed across the study area (Fig. 1).

Within each site, we evenly distributed four plastic trays (Sterilite, 34.60 × 20.96 × 12.38 cm), each containing 100 first-instar *Ae. albopictus* larvae and 1l of leaf infusion. *Aedes albopictus* were from a laboratory colony obtained from the Centers for Disease Control (Atlanta, GA, USA) originating from Keyport, NJ, USA in 1995 (strain ATM-NJ95) [20] and maintained following standardized protocols. Leaf infusion was prepared as described in Murdock et al. [16]. Briefly, 80 g live oak (*Quercus virginiana*) leaves and 3 g of 1:1 yeast:albumin mixture were infused in deionized water. Trays were screened with a fine mesh, placed in a wire cage to deter wildlife, covered with clear plastic vinyl to keep rainwater from entering, and placed in full shade. We added deionized water to trays after two weeks to maintain a total water volume at 1l. We placed data loggers [Monarch Instruments, Amherst, NH, USA: Radio Frequency Identification (RFID) Temperature Track-It Logger] in vegetation next to each tray, approximately 0.9 m above the ground. Data loggers recorded instantaneous temperature and relative humidity at ten minute intervals throughout the study period. Data loggers were also placed in the trays to measure the larval, aquatic temperature, however three and 17 loggers (of 36) failed due to water damage in the summer and autumn, respectively. Of loggers that did not fail during the experiment, water temperatures were highly correlated with ambient temperatures (*ρ* = 0.929); thus, only ambient temperatures are used as an approximation of larval environmental temperature. Sites were visited daily to collect emerging adults until all larvae had emerged or died (Summer Replicate: August 1 to September 3, 2016, Autumn Replicate: September 26 to November 8, 2016). We quantified the total number of adults emerging per day, and recorded the sex and wing length of each emerged adult. Adult females were collected to use in vector competence assays.

### Dengue virus *in vitro* culturing and mosquito infections

DENV-2 stock was obtained from the World Reference Center for Emerging Viruses and Arboviruses at the University of Texas Medical Branch (PRS 225 488, originally isolated from human serum in Thailand in 1974 [21]). We propagated virus by inoculating Vero (African green monkey kidney epithelial) cells with a low MOI infection. Virus-containing supernatant was harvested when the cells exhibited more than 80% cytopathic effect. Supernatant was cleared of cell debris by centrifugation (1000× *g*, 1 min), aliquoted into cryo-vials, and stored at -80 °C. We quantified viral titers of virus stock using TCID-50 assays, calculated by the Spearman-Karber method [22, 23]. When mixed 1:1 with the red blood cell mixture, the final concentration of virus in the blood meal was 3.540 × 10^6^ *TCID*_50_/ml.

Adult mosquitoes were collected as they emerged from trays, aggregated by site, and stored in reach-in incubators at 27 ± 0.5 °C, 80 ± 5% relative humidity, and a 12:12 h light:dark photocycle. To ensure infected mosquitoes were of a similar age, mosquitoes were pooled into cohorts of 4–6 days-old in the summer and 4–9 days old in the autumn (due to slower and more asynchronous emergence rates). Mosquitoes were allowed to mate and fed *ad libitum* with a 10% sucrose solution. Forty-eight hours prior to infection, the sucrose was replaced with deionized water, which was then removed 12–14 h before infection to encourage feeding. Infectious blood meals were administered to mosquitoes through a water-jacketed membrane feeder and consisted of 47% human red blood cells washed in DMEM (v/v), 1% sucrose (w/v), 20% FBS (v/v), 5 mM ATP, and 33% DMEM medium combined with 1 ml of virus stock [24]. Blood-fed female mosquitoes were then maintained as described above for the duration of the experiment.

For a mosquito to become infectious, arboviruses must pass through multiple tissues that impose significant barriers to infection, namely the midgut and salivary glands [25]. Therefore, we assessed mosquitoes for infection, dissemination, and infectiousness through salivation assays and tissue dissections 21 days post-infection [26]. First, mosquitoes were cold-anesthetized and immobilized by removing their legs and wings. Wings were mounted on a glass slide to measure wing length from the distal end of the alula to the apex of the wing *via* a dissecting scope and micrometer. The proboscis of each female was then inserted into a sterile pipette tip containing 10–20 μl of FBS (with 3 mM ATP and red food coloring) and allowed to salivate on a plate kept at 27 °C for 15 min, after which the salivation media was expelled into 500 μl of DMEM and stored at -80 °C. After salivation, we removed the head of each individual and stored the body and head separately at -80 °C.

To determine variation in the proportion of mosquitoes that become infected (bodies positive for virus), disseminated (heads positive for virus), and infectious (saliva positive for virus), we used cytopathic effect (CPE) assays to test for the presence of virus in each collected tissue [23]. Individual bodies and heads were homogenized in 500 μl of DMEM and centrifuged at 2500 *rcf* for 5 min. Two-hundred microliters of homogenate was added to Vero cells in a solution of DMEM (1% pen-strep, 5% FBS by volume) in a 24-well plate and kept at 37 °C and 5% CO_2_. Salivation media was thawed and plated on Vero cells as above. After 5 days, Vero cells were assessed for presence of DENV-2 *via* CPE assays. Samples were identified as positive for virus if CPE was present in the well.

All infection work was conducted in an arthropod containment level 2 (ACL-2) facility at the University of Georgia in the College of Veterinary Medicine. The physical space as well as experimental protocols have been reviewed and approved by the University of Georgia Office of Biosafety (2015-0038). Briefly, all DENV-2 exposed mosquitoes were counted initially and throughout the experiment, housed in secondary containment cages, and handled in a glove box and on ice when they were removed from secondary containment for forced salivations. All virus assays were also conducted in a biosafety cabinet in our biosafety level II (BSL-2) facility. Finally, we used designated and approved secondary containment to transport virus or infected tissues between our ACL-2 and BSL-2.

### Intrinsic growth rates (*r*’) and vectorial capacity (*VC*)

We calculated the per capita population growth rate per tray following Livdahl & Sugihara [27] (Eqn. 1):

1 $$ {r}^{\hbox{'}}=\frac{\ln \left(\frac{1}{N_0}{\Sigma}_x{A}_xf\left(\overline{w_x}\right)\right)}{D+\frac{\Sigma_x{xA}_xf\left(\overline{w_x}\right)}{\Sigma_x{A}_xf\left(\overline{w_x}\right)}} $$

Following Livdahl & Sugihara [27], we assume N_0_ to represent the initial number of females before accounting for mortality during the larval stage. This enables the mortality rate to be included *via* the summed A_x_/N_0_ parameter. Setting N_0_ equal to the number of emerged mosquitoes would imply a 100% larval survival rate, which was not the case in our study. Unfortunately, we cannot identify the sex of first-instar larvae, and must assume a ratio within the initial cohort. While we do record the proportion of emerged mosquitoes, this represents those that have survived the larval environment until emergence, and, given our findings regarding the effects of the larval microclimate on larval survival, may not be representative of the initial cohort. In our study, males emerged 1–3 days earlier than females in the summer, and up to a week earlier than females in the autumn. This additional time in the larval environment could have exposed female mosquitoes to stressful temperatures and lower resource concentrations than the male mosquitoes that emerged earlier, resulting in lower emergence rates. Eggs used in the experiment were drawn from a laboratory colony which is known to have approximately a 50:50 male:female sex ratio. Therefore, we used this value in (50% of the larvae, 50) our calculations. The other parameters are defined as follows: *A*_*x*_ is the number of mosquitoes emerging on day *x*, *D* is the time to reproduction following emergence (assumed to be 14 days [28]), and $f\left(\overline{w_x}\right)$ is fecundity as a function of mean wing size on day *x* ($\overline{w_x}$; Eqn. 2). This relationship is assumed to be linear and calculated *via* Lounibos et al. [29] (Eqn. 2):

2 $$ f\left(\overline{w_x}\right)=-121.240+\left(78.02\times \overline{w_x}\right) $$

While it is possible to reason how changes in each parameter will result in carry-over effects that individually affect disease transmission, determining the overall net effect and magnitude of the change is less straightforward. Therefore, we calculated the vectorial capacity (*VC*; Eqn. 3) for each site and season using a modified temperature-dependent dengue calculation defined in Mordecai et al. [30] to create a quantitative estimate of the influence of carry-over effects on disease transmission. Using the experimental mean for field-derived parameters affected by carry-over effects (fecundity and vector competence), we calculated an additional site-level *VC* to serve as an estimate of this value when *not* accounting for site-specific carry-over effects.

3 $$ VC(T)=\frac{a{(T)}^2b(T)c(T){e}^{-e(T)/ EIR(T)} EFD(T){p}_{EA}(T) MDR(T)}{\mu {(T)}^2} $$

Here, mosquito traits are a function of temperature (*T*), as described in Table 1. Site-level *VC* was calculated using a combination of traits empirically measured in this study and traits estimated from thermal response models as described in Mordecai et al. [30].

**Table 1:** Parameters used in the *VC* calculation. Parameters sourced from Mordecai et al. [30] were mathematically estimated at a constant temperature of 27 °C, the temperature at which our adult mosquitoes were housed. Parameters that included carry-over effects are starred

Parameter	Definition	Source	Mean (Range)^a^
*a*(*T*)	Per-mosquito bite rate	Mordecai et al. [30]	0.294 (-)
*b*(*T*)*c*(*T*)*	Vector competence	Present study	0.107 (0–0.353)
*μ*(*T*)	Adult mosquito mortality rate	Mordecai et al. [30]	0.011 (-)
*EIR*(*T*)	Extrinsic incubation rate (inverse of extrinsic incubation period)	Mordecai et al. [30]	0.196 (-)
*EFD*(*T*)*	No. of eggs produced per female mosquito per day	Present study	18.678 (15.260–22.800)
*p*_*EA*_(*T*)	Egg-to-adult survival probability	Present study	0.485 (0.090–0.775)
*MDR*(*T*)	Larval development rate	Present study	0.056 (0.027–0.087)

^a^Mean and range are shown for each parameter, except for those calculated at a constant adult environmental temperature which did not change

The bite rate (*a*(*T*)), adult mosquito mortality rate (*μ*(*T*)), and extrinsic incubation rate (*EIR*(*T*)), were calculated for mosquitoes at a constant 27 °C using temperature-dependent functions from Mordecai et al. [30], to match the adult environment used in the experiment. Vector competence (*b*(*T*)*c*(*T*)) was calculated as the proportion of infectious mosquitoes per site as determined by our DENV-2 infection assays. Conventionally, vector competence is the product of the proportion of mosquitoes that become infected after biting an infected human and the proportion of bites by infectious mosquitoes that infect humans. Our estimate, the proportion of infectious mosquitoes as measured by CPE assays, is the same as the product of the proportion of mosquitoes that become infected following an infectious blood meal and the proportion of infected mosquitoes that have DENV-2 virus particles in their saliva. With this formulation we are assuming that all infectious bites result in human infection, as we are not directly measuring dengue infection outcomes in humans (i.e. effects of human immunity on DENV infection). The number of eggs produced per female per day (*EFD*(*T*)) was calculated by estimating fecundity from average female wing length following Eqn. 2, and then dividing this by the expected lifespan of mosquitoes (1/*μ*). The egg-to-adult survival probability (*p*_*EA*_(*T*)) was defined as empirically measured egg-to-adult survival probability (the average proportion of adult female mosquitoes emerging per site). The mosquito immature development rate (*MDR*(*T*)) was calculated as the inverse of the mean time to emergence for female mosquitoes per site, resulting in a daily rate of development. To estimate bias introduced by not including carry-over effects, we compared our site-level calculated *VC* to one calculated using the experimental grand mean for site-level *EFD* and *bc*. All other parameters were the same across the two models.

### Statistical analysis

We used linear mixed models (LMMs) to explore if microclimate (i.e. mean, minimum, maximum, and daily ranges of temperature and relative humidity), larval development rate (1/days to emergence), female body size, and per capita growth rate differed across land class and season. Egg-to-adult survival (the proportion of adult females emerging per tray) and metrics of vector competence (i.e. infection, dissemination, and infectiousness) were fit using generalized linear mixed models (GLMMs) with binomial distributions and *logit* links. In all models, fixed effects included land class, season, and their interaction, with site as a random effect. The effect of body size on infection dynamics was also explored at the level of the individual mosquito, fitting a binomial GLMM including wing size as a fixed effect and site as a random effect. Vectorial capacity was calculated at the site-level, and so did not require site to be included as a random effect. We therefore used a regression model to estimate the effect of land class, season, and their interaction on site-level vectorial capacity.

To confirm the relationship between the categorical variables of land use and season and temperature, we fit additional models containing mean temperature as a covariate to the residuals of the original models including season and land use as fixed effects. This test explored if there was additional variation in the response variable due to temperature that was not explained by land class and season. To explore if the effect of temperature differed across season, we fit individual models to the above response variables including mean temperature as averaged across each season (e.g. summer or autumn) as a covariate, using the same distributions and link functions. For egg-to-adult survival, larval development, body size, and the per capita growth rate, mean temperature was calculated over each individual season (e.g. summer and autumn) at the tray level, and site was included as a random effect. Because mosquitoes were pooled by site for infection assays, temperature was aggregated to the site level and no random effects were included for analyses of infection metrics and *VC.*

All analyses were conducted with respect to the female subset of the population, as they are the subpopulation responsible for disease transmission. In the case of data logger failure (*n* = 3), imputed means from the site were used to replace microclimate data. In the case of trays failing due to wildlife tampering (two urban and one suburban in the autumn replicate), collected mosquitoes were used for infection assays, but trays were excluded from demographic analyses. For all mixed-models, significance was assessed through Wald Chi-square tests (*α* = 0.05) and examination of 95% confidence intervals. Pearson residuals and Q-Q plots were visually inspected for normality. All mixed models were fit using the *lme4* [31] package in R v. 3.5.0 [32]. Code to run analyses and create figures is deposited on figshare.

## Results

### Effects of land class and season on microclimate

**Fig. 2:**
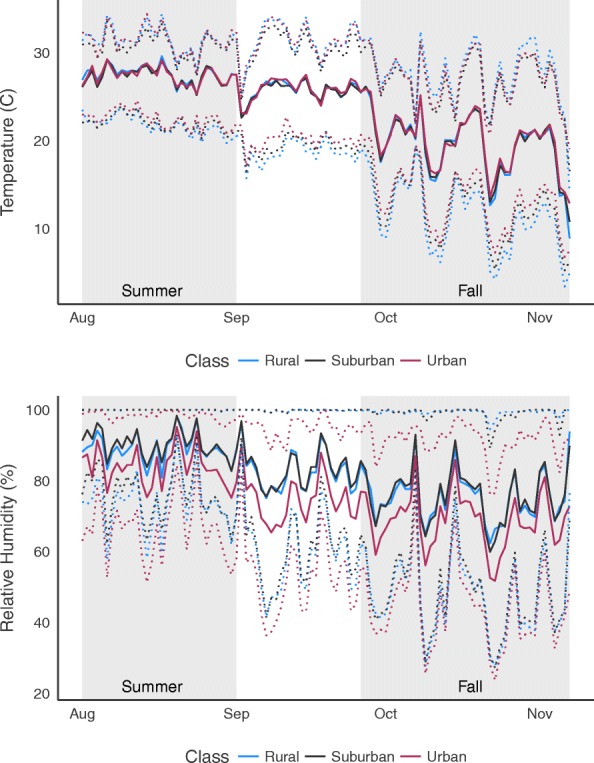
Temperature and relative humidity across season and land class. The solid line represents the mean temperature and relative humidity across trays in each land class. The dotted lines represent the mean minimum and maximum temperature and relative humidity across trays in each land class

We found that microclimate profiles differed significantly across both season and land class (Fig. 2, Table 2). In general, temperatures were warmer in the summer and on urban sites, replicating what was found in a prior study in this system [16]. We did observe a significant interaction between season and land use on the mean daily minimum temperature and diurnal temperature range, with no effect of land use on these response variables in the summer. Urban sites in the autumn were characterized by significantly higher daily average minimum temperature and smaller diurnal temperature range relative to rural sites (Table 2). Mean relative humidity was higher in the summer than the autumn: [mean (95% CI)], summer: 87.93% (86.33–89.54 %); autumn: 73.32% (71.72– 74.92%). In the summer, minimum and mean relative humidity was significantly lower on urban sites compared to rural and suburban sites (Table 2). A similar trend was seen in the autumn, with urban sites having lower mean relative humidity compared to other land classes, but no difference in minimum relative humidity (Table 2).

**Table 2:** Mean microclimate values (95% confidence intervals) across season and land class. Letters represent significant differences as measured by pairwise comparison using Tukey’s multiple comparison of means, adjusting for significance with the Holm-Bonferroni method

Microclimate variables	Summer			Autumn		
	Rural	Suburban	Urban	Rural	Suburban	Urban
Minimum temperature (°C)	21.73 (20.93–22.53)^a^	22.00 (21.20–22.80)^a^	22.67 (21.87–23.47)^a^	11.03 (10.23–11.83)^b^	12.23 (11.43–13.03)^bc^	13.41 (12.61–14.21)^c^
Mean temperature (°C)	27.58 (27.13–28.02)^a^	27.38 (26.94–27.83)^a^	27.45 (27.01–27.90)^a^	19.45 (19.01–19.89)^b^	19.55 (19.10–19.99)^b^	19.95 (19.51–20.40)^b^
Maximum temperature (°C)	31.53 (30.76–32.30)^a^	30.86 (30.09–31.63)^a^	31.40 (30.63–32.17)^a^	27.57 (26.80–28.34)^b^	26.58 (25.81–27.35)^b^	26.85 (26.08–27.62)^b^
Daily temperature range (°C)	9.81 (8.51–11.11)^a^	8.86 (7.56–10.16)^a^	8.73 (7.43–10.03)^a^	16.54 (15.24–17.84)^b^	14.35 (13.05–15.65)^bc^	13.44 (12.14–14.74)^c^
Minimum relative humidity (%)	73.49 (69.39–77.59)^ab^	76.29 (72.19–80.39)^a^	67.40 (63.30–71.50)^b^	47.68 (43.58–51.78)^c^	48.84 (44.74–52.94)^c^	44.14 (40.04–48.24)^c^
Mean relative humidity (%)	89.01 (86.23–91.780)^ab^	90.38 (87.61–93.16)^a^	84.43 (81.66–87.20)^b^	75.39 (72.61–78.16)^c^	75.57 (72.79–78.34)^c^	69.01 (66.23–71.78)^d^
Maximum relative humidity (%)	99.95 (97.14–100.00)^a^	99.98 (97.17–100.00)^a^	98.38 (95.58–100.00)^a^	99.36 (96.56–100.00)^b^	98.93 (96.12–100.00)^b^	91.77 (88.97–94.58)^c^
Daily humidity range (%)	26.46 (22.07–30.85)^a^	23.69 (19.30–28.08)^a^	30.98 (26.59–35.37)^a^	51.69 (47.29–56.08)^b^	50.09 (45.70–54.49)^b^	47.63 (43.24–52.02)^b^

### Direct and carry-over effects of land class and season on population growth

**Fig. 3:**
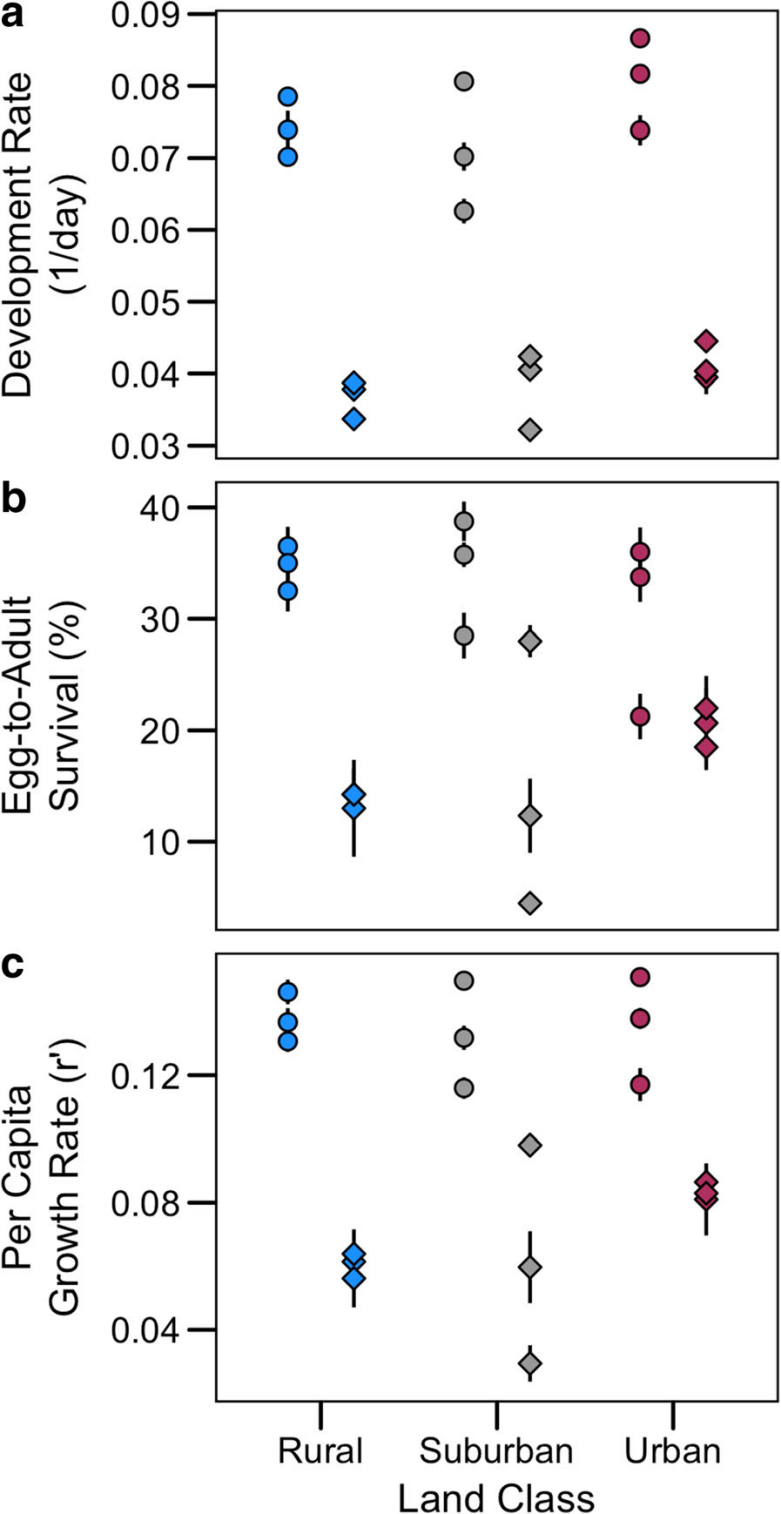
Demographic rates of mosquitoes across season and land class. Female larval development rate (**a**), egg-to-adult survival (**b**), and per capita population growth rate (**c**) across the summer (circle) and autumn (diamond) trials and rural, suburban, and urban land classes. Points represent site-level means (e.g. the mean of all four trays within a site for each season) with standard error bars. Some standard error bars are not visible because they are small enough to be obscured by the point

Of the 3600 first-instar larvae placed in each season, a total of 2595 and 1128 mosquitoes emerged in the summer and autumn, respectively. The total female egg-to-adult survival per tray was significantly higher in summer than autumn [Table 3, mean (binomial asymptotic 95% CI), summer: 0.670 (0.598–0.735); autumn: 0.297 (0.235–0.366)], but did not differ across land class (Fig. 3a, Table 3). The mean rate of larval development per tray was significantly different between summer and autumn (Fig. 3b, Table 3), with daily mean ± SE development rates of 0.074 ± 0.002 and 0.0387 ± 0.002, respectively. There were no significant differences in larval survival or development rates across land class. We did not observe a significant carry-over effect of land class or season on mosquito wing size, however there was a significant interaction between the two (Table 3). We found a significant difference in wing size across season for mosquitoes on rural sites only, with larger bodied mosquitoes in the summer (mean ± SD 2.451 ± 0.211 mm), than the autumn (2.300 ± 0.202 mm). While urban mosquitoes tended to be larger in the autumn, and suburban mosquitoes tended to be larger in the summer, these effects were not significant.

**Table 3:** GZLM model results of land class, season and their interaction on demographic and infection rates. Significance was assessed *via* Wald Chi-square tests (α = 0.05) and there was no evidence that data failed to meet assumptions of normality

Variable of interest	Class			Season			Class*Season		
	*df*	*χ* ^2^	*P*-value^a^	*df*	*χ* ^2^	*P*-value^a^	*df*	*χ* ^2^	*P*-value^a^
Egg-to-adult survival	2	0.0361	0.982	1	61.129	**< 0.001**	2	5.891	0.0526
Development rate	2	3.847	0.1461	1	597.51	**< 0.001**	2	3.108	0.2114
Wing length	2	0.8348	0.6587	1	2.7937	0.0946	2	14.748	**< 0.001**
Per capita growth (r’)	2	0.667	0.717	1	219.84	**< 0.001**	2	2.622	0.23
Infection	2	18.168	**< 0.001**	1	12.271	**< 0.001**	2	1.985	0.371
Dissemination	2	14.253	**< 0.001**	1	14.909	**< 0.001**	2	0.941	0.625
Infectiousness	2	1.105	0.575	1	3.63	0.057	2	0.302	0.860
Vectorial capacity	2	0.161	0.922	1	5.721	**0.017**	2	0.905	0.636

^a^Significant effects at the 0.05 level are indicated in boldface

After incorporating the number of adult females emerging per day, the day of emergence, and their body size into the per capita growth rate equation (Eqn. 1), we found that the estimated per capita growth rate was higher in the summer season than the autumn season (Fig. 3c, Table 3, mean ± SE, summer: 0.135 ± 0.005; autumn: 0.068 ± 0.006) with no difference across land class. The effect of temperature within a season was only significant for egg-to-adult survival, and differed in direction across season (mean β ± SE, summer: -0.328 ± 0.148; autumn: 0.368 ± 0.135, Additional file 1: Table S1). This mirrors a trend for the effect of land class on egg-to-adult survival to differ across season (Table 3). When controlling for land class and season, temperature explained no additional variation for any response variable (Additional file 1: Table S2).

### Carry-over effects of land class and season on vector competence

**Fig. 4:**
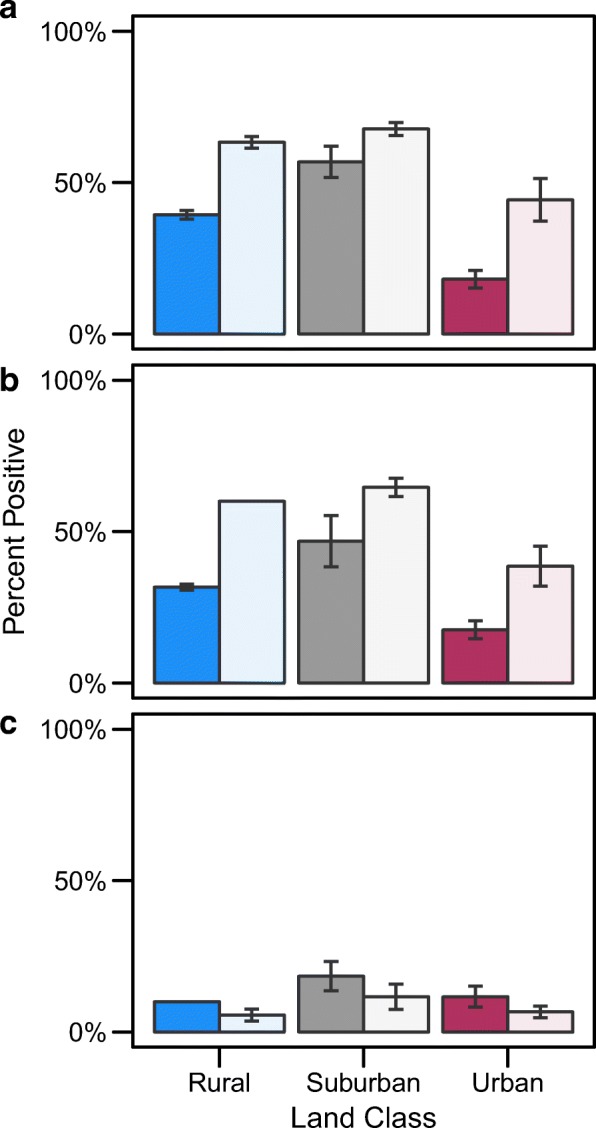
Infection rates of mosquitoes across season and land class. Rates of infection (**a**), dissemination (**b**), and infectiousness (**c**) of dengue in female mosquitoes at 21 days post-infection across the summer (dark fill) and autumn (light fill) trials and rural, suburban, and urban land classes. Mean site-level values are plotted with error bars representing standard error (*n* = 3)

A total of 319 female mosquitoes were assessed for infection status, 20 per site in the summer and varying numbers per site in the autumn due to lower emergence rates (sample sizes reported in Table 4). Carry-over effects of the larval environment on infection status were limited to infection and dissemination rates. We found that land class and season did significantly impact the probability of a mosquito becoming infected and disseminating dengue infection (Table 3). Both metrics were higher in the autumn compared to the summer replicate, with urban sites having the lowest infection and dissemination rates across both seasons (Fig. 4a, b). While there was a trend for a higher proportion of mosquitoes becoming infectious in the summer (Fig. 4c), this was not significant (*χ*^2^ = 3.63, *P* = 0.057). The probability of becoming infectious did not differ across land class, nor season (Fig. 4c, Table 3), despite the higher probability of mosquito infection and dissemination in the autumn, and on suburban and rural sites. Similarly, there was no effect of temperature on any infection metric within a season (Additional file 1: Table S1), and temperature did not explain any additional variation after controlling for land class and seasons (Additional file 1: Table S2). This suggests that the ability of virus to escape the midgut and invade the salivary glands differs in adults reared in the summer *vs* the autumn and across land class, with a higher proportion of dengue infected mosquitoes becoming infectious in the summer and on urban sites (Table 4, (*χ*^2^ = 13.65, *P* < 0.001). We also found the probability of infection to decline with increasing body size (*χ*^2^ = 4.776, *P* = 0.0289, although there was no evidence for a relationship between body size and the probability of dissemination or infectiousness.

**Table 4:** Dengue infection rates. The rates of infection (mosquitoes with dengue positive bodies), dissemination (infected mosquitoes with dengue positive heads) and infectiousness (infected mosquitoes with dengue positive saliva) across season and land class. Raw numbers of positive samples are shown with the denominator in parentheses

Season	Land class	No. infected (*n*)	No. disseminated (*n*)	No. infectious (*n*)
Summer	Rural	22 (56)	19 (60)	6 (60)
	Suburban	32 (57)	26 (57)	10 (57)
	Urban	10 (51)	10 (53)	7 (53)
Autumn	Rural	32 (50)	30 (50)	3 (47)
	Suburban	28 (43)	25 (41)	3 (43)
	Urban	26 (59)	22 (57)	4 (59)

*Abbreviation*: *n* sample size

### Integrating direct and carry-over effects into estimates of transmission potential

**Fig. 5:**
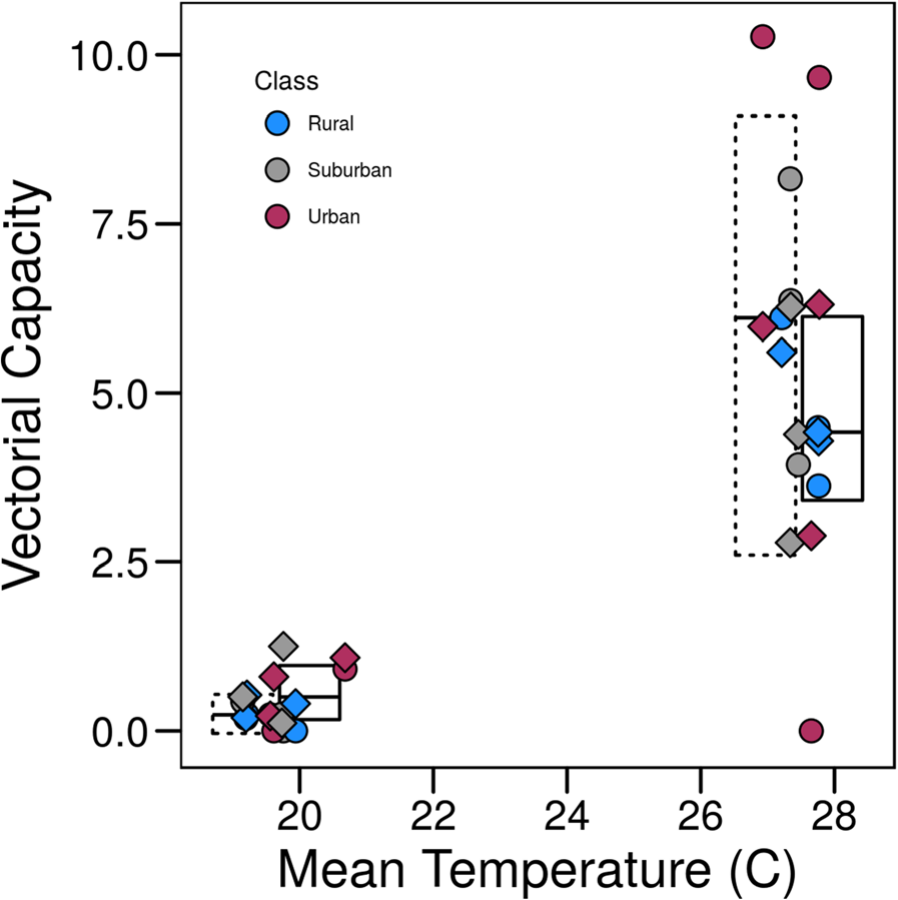
The effect of larval temperature on predicted vectorial capacity at the site and seasonal level. Points represent site-level *VC* calculations for field based (circle) and grand mean (diamond) calculations, with colors representing the sites’ land class. Boxes represent mean ± SD per calculation type (field based: dotted *vs* grand mean: solid) and season (summer *vs* autumn)

We found *VC* to be higher in the summer (mean ± SE: 5.847 ± 0.768) than the autumn (0.252 ± 1.097) (Fig. 5, Table 3). In the summer season, there was a trend for *VC* to increase with increasing urbanization (Fig. 5). This trend was not significant, however, given the small sample size (*n* = 9) and the disproportional impact of having no infectious mosquitoes at one site, resulting in a value of *VC* = 0 for one sample. There was no effect of temperature on *VC* within a season (Additional file 1: Table S1), and temperature did not explain any additional variation after controlling for land class and season. When comparing *VC* calculations using field-based or grand mean estimates of *EFD* and *bc*, we found that the effect of land class and season were not significantly different (land class: *χ*^2^ = 0.381, *P* = 0.826), season: *χ*^2^ = 1.408, *P* = 0.235), suggesting that the omission of carry-over effects in calculations did not lead to biased estimates of relative *VC* in different seasons or land classes. However, the use of the grand mean did lead to an underestimate of *VC* on some suburban and urban sites in the summer, with a two-fold decrease in predicted *VC* (Fig. 5, Additional file 2: Figure S1). The calculated *VC* for rural sites in the summer and across all land classes in the autumn more closely resembled the grand mean calculated *VC*.

## Discussion

Mathematical models of mosquito-borne disease rarely include mosquito larval stages [15], and of those that do, few include the influence of carry-over effects on important mosquito life-history traits (but see [33]). This is likely because there are relatively few empirical studies parameterizing carry-over effects in mosquito-pathogen systems [2], and most are laboratory studies conducted across a wider range of temperatures than those seen in the field. Here, we demonstrate that fine-scale differences in larval microclimate across land class and season generate carry-over effects on adult fecundity and vector competence for DENV-2. When integrated into a model of vectorial capacity, we find that vectorial capacity differs across season, but not land class. Further, failure to account for site-specific carry-over effects across urban land classes results in biased estimates of DENV-2 transmission potential, underestimating potential disease transmission in urban areas.

The subtle heterogeneity in microclimate we observed across season resulted in significantly different predicted population growth rates through its effects on demographic traits. Daily mean temperatures (25.43 °C) across all sites in the summer were closer to the predicted thermal optimum of *Ae. albopictus* (24–25 °C) [30] than in the autumn (17.69 °C), leading to higher egg-to-adult survival rates. We also observed more rapid larval development rates in the summer relative to the autumn. This is likely due to the strong positive relationship observed between development rates and mean larval temperature, as the metabolic rate of mosquitoes will increase with warming temperatures [3]. Temperature explained no additional variation in any response variable after accounting for land class and season, suggesting that our coarser characterizations of land class and season contain the temperature variation necessary to predict changes in demographic and infection rates. Additionally, we only found an effect of temperature within a season for egg-to-adult survival (Additional file 1: Table S1). While we did not find a significant influence on many traits, our trends do agree with a previous study in this system that found lower egg-to-adult survival on urban sites [16]. The variation in mean temperature across land class in our study was very small (< 1 °C), and we expect these relationships would be magnified in mega-cities that can have urban heat island effects of up to 6 °C [34].

Surprisingly, we found no main effect of land class or season on female mosquito body size, despite the difference in temperatures across season. Following allometric temperature-size relationships of ectotherms, warmer larval temperatures should lead to smaller-bodied mosquitoes [35]. However, contrary to predictions generated from the allometric temperature-size relationship, we observed mosquitoes on rural sites to be larger in the summer despite the fact that all land classes were cooler in the autumn relative to the summer. Our results contrast with many laboratory studies that have found a negative relationship between rearing temperature and mosquito body size (*Ae. albopictus* [36], *Culex tarsalis* [37], *Anopheles gambiae* [38]). However, these studies all used a constant temperature treatment, while mosquitoes in our field-based study experienced fluctuating temperatures. Among studies using fluctuating temperatures, there is mixed evidence for a relationship between rearing temperature and mosquito body size [16, 39]. Larger temperature fluctuations at the more extreme temperatures (cool and warm) can lead to counterintuitive effects of temperature on organismal traits if these temperatures approach or cross the thermal maximum or minimum (at which trait performance is zero) and induce thermal stress [40, 41]. Rural sites in the autumn did experience a larger average diurnal range of temperatures than in the summer, suggesting this differential effect of temperature fluctuations at thermal extremes could be acting on body size. Our findings demonstrate that, while the use of fluctuating temperatures in studies of mosquito life-history traits is relatively new, these fluctuations can have significant impacts on mosquito ecology and should be integrated in laboratory-based studies of mosquito vectors to more closely approximate field conditions.

Our results agree with laboratory studies in other arboviral systems (chikungunya [42], yellow fever [42], and Rift Valley fever [43]) that found cool larval environmental temperatures to enhance arbovirus infection relative to warmer larval environments. Studies in the *Ae. albopictus*-dengue virus system have also found that low larval temperatures enhance mosquito susceptibility to viral infection, although this is dependent on larval nutrition [10] and the stage of the infection (i.e. midgut *vs* dissemination *vs* saliva) [9]. While we found infection and dissemination of DENV-2 to decrease with increasing temperatures across season and land class, there was no effect on viral presence in the saliva, suggesting carry over effects due to microclimate variation may alter the overall efficiency of dengue infection. Thus, even though a smaller proportion of mosquitoes reared on urban sites and in the summer became infected and disseminated infection, these mosquitoes were more likely to become infectious, resulting in no net difference in overall vector competence across land class and season. Larval environmental temperature may differentially impact later stages of viral infection (i.e. salivary gland penetration) compared to earlier stages (i.e. midgut escape) through effects on mosquito physiology and immunity, as well as on important tissue barriers to infection [4, 42, 44, 45]. Further, our study considered only DENV-2, and other arboviruses and mosquito-borne disease are likely influenced by the mosquito’s larval environment differently.

Current models of vector-borne disease focus primarily on direct effects of environmental variables on mosquito densities and disease transmission and rarely include the effects of the larval stage, either directly or *via* carry-over effects [15]. While we found carry-over effects due to seasonal and urban environments to have a significant impact on DENV-2 infection and dissemination, we found no net effects on saliva positivity for the virus. Therefore, when incorporating parameters into calculations of vectorial capacity, we did not find a significant difference in predicted vectorial capacity due to land class. However, we did find *VC* to be higher in the summer relative to the autumn, driven by differences in demographic rates such as larval survival and development rates, rather than differences in adult vector competence. Unfortunately, given the logistical limitations imposed by a field experiment setting, we were unable to measure additional life-history traits important for disease transmission in conjunction with vector competence. Laboratory studies have found that factors such as adult longevity [46], biting rate [47], and pathogen extrinsic incubation period [48, 49] are also be impacted by carry-over effects. For example, warmer larval temperatures correspond with decreased adult longevity in mosquitoes [46], and including this relationship could mediate the seasonal differences in *VC* found in our study, with decreased adult longevity in the summer corresponding to decreased *VC*. Less is known about traits specific to transmission such as biting rate and EIP, which have only been investigated in response to larval diet and competition [47–49]. Carry-over effects of the larval environment can act on multiple adult phenotypes, often in conflicting ways, and the net effect of this on disease transmission has yet to be fully explored.

Our study was further limited by the difficulties in obtaining appropriate sample sizes. While semi-field experiments incorporate more realistic variation in environmental temperature than laboratory experiments, they require additional space and travel time in order to distribute replicates in a manner that meets assumptions of independence across sites. Given the size of our study area, nine was the maximum number of sites that it was possible to visit daily. Unexpectedly low emergence rates of mosquitoes in the autumn further reduced the sample size of mosquitoes that could be used in infection assays. Despite this limitation, we did find significant differences in mosquito demographic rates across season and in infection and dissemination rates across land class, suggesting that site-specific characteristics can directly and indirectly impact vector-borne disease dynamics. Yet, due to the low replication across sites, these results must be interpreted conservatively.

Carry-over effects are not simply limited to microclimate, and can result due to variation in larval nutrition [47], intra- and interspecific densities [50], and predation [33] in mosquito systems. Further, abiotic and biotic factors will likely interact to influence carry over effects [10, 51], and this interaction could be scale-dependent [52]. For example, biotic processes are predicted to be more important at local geographical scales, while abiotic processes dominate at regional geographical scales in species distribution models [53]. Future exploration of the scale-dependent contribution of different environmental factors and their interactive influence on both direct and carry-over effects is needed to improve models predicting the distribution of mosquito vector species, mosquito population dynamics and disease transmission.

## Conclusions

We found fine-scale variation in microclimate across season and urban land class to shape *Ae. albopictus* population dynamics and DENV-2 transmission potential through direct effects on larval survival and development rates, and indirectly through carry-over effects on vector competence and fecundity. Although sample sizes were limited, our study indicates the potential effects that site-specific environments can have on mosquito demographics and infection dynamics. DENV-2 infection and dissemination rates were higher in mosquitoes from rural and suburban land classes than urban ones, and were higher in the autumn compared to the summer. However, there was no difference in overall infectiousness. Therefore, the seasonal differences in *VC* we observed were due to the direct effects of the larval environment on egg-to-adult survival and development rates, rather than carry-over effects. When comparing *VC* to a calculated *VC* that did not account for site-specific carry-over effects, we found that not accounting for carry-over effects results in an underestimate of predicted *VC* in suburban and urban sites in the summer, and an overestimate in the autumn. The interaction between the larval and adult environments, mediated by carry-over effects, could have complex consequences for adult phenotypes relevant to disease transmission for mosquitoes as well as other organisms. Given the devastating impact of disease in other species with complex life histories (e.g. chytridiomycosis in amphibians), carry-over effects in disease transmission are important, though understudied, mechanisms that must be better understood to control disease spread. Incorporating relationships between carry-over effects and organismal life-history traits into statistical and mechanistic models will lead to more accurate predictions on the distributions of species, population dynamics, and the transmission of pathogens and parasites. Mosquito-borne disease incidence is spatially heterogeneous in urban areas [54], and a better understanding of both the larval and adult environments, including their interaction, could improve the accuracy of fine-scale predictions of disease incidence across a city.

## Additional files

Additional file 1: **Table S1.** Effect of temperature within a season. Model results from GZLMs estimating the effect of temperature within a season. Binomial models were fit with a logit-link function. Except for those models predicting infection metrics and vector competence, site was included as a random effect. **Table S2.** Additional variation in residuals explained by temperature. Model results from fitting temperature to residuals of original models (land class × season) for each response variable. In all models, temperature did not explain any additional variation, as evidenced by low mean sum of squares and *F*-statistics. (PDF 18 kb)

Additional file 2: **Figure S1.** Bias in *VC* due to not accounting for site-level carry-over effects across land class and season. (PDF 7 kb)
