# Supplementary material for: Carry-over effects of urban larval environments on the transmission potential of dengue-2 virus
Source: Parasit Vectors. 2018 Jul 17;11:426. doi: 10.1186/s13071-018-3013-3 (PMC6050736; doi:10.1186/s13071-018-3013-3)
Supplement: Supplementary file 1 — Table S1. Effect of temperature within a season. Model results from GZLMs estimating the effect of temperature within a season. Binomial models were fit with a logit-link function. Except for those models predicting infection metrics and vector competence, site was included as a random effect. Table S2. Additional variation in residuals explained by temperature. Model results from fitting temperature to residuals of original models (land class × season) for each response variable. In all models, temperature did not explain any additional variation, as evidenced by low mean sum of squares and F-statistics. (PDF 18 kb) [file 13071_2018_3013_MOESM1_ESM.pdf]

S1 Table. Effect of temperature within a season. Model results from GZLMs estimating the effect of temperature within a season. Binomial models were fit with a logit-link function. Except for those models predicting infection metrics and vector competence, site was included as a random effect.

		β	**df**	χ^2	**F-value**	**p-value**
Summer	Survival	-0.328	1	4.943	-	**0.026**
	Development	0.000	1	0.007	-	0.936
	Wing Length	0.060	1	2.343	-	0.126
	Growth	-0.006	1	0.774	-	0.379
	Body Inf	-0.348	1	0.240	-	0.624
	Head Inf	-0.346	1	0.223	-	0.637
	Sal Inf	-0.488	1	0.580	-	0.446
	VC	-5.484	1	-	2.275	0.175
Fall	Survival	0.368	1	7.441	-	**0.006**
	Development	0.002	1	2.554	-	0.110
	Wing Length	0.037	1	1.644	-	0.200
	Growth	0.011	1	1.717	-	0.190
	Body Inf	-0.211	1	0.378	-	0.539
	Head Inf	-0.346	1	1.032	-	0.310
	Sal Inf	-0.932	1	1.276	-	0.259
	VC	0.295	1	-	2.173	0.184

Table S2. Additional variation in residuals explained by temperature. Model results from fitting temperature to residuals of original models (land class x season) for each response variable. In all models, temperature did not explain any additional variation, as evidenced by low mean sum of squares and F-statistics.

	**df**	**Mean Sq**	**F value**
Survival	1	1.948	0.302
Development	1	0.000	0.324
Wing Length	1	0.002	0.055
Growth	1	0.000	0.000
Body Inf	1	0.795	0.617
Head Inf	1	0.156	0.124
Sal Inf	1	0.079	0.132
VC	1	0.031	0.006
