# Supplementary figures and images for: Carry-over effects of urban larval environments on the transmission potential of dengue-2 virus
Source: Parasit Vectors. 2018 Jul 17;11:426. doi: 10.1186/s13071-018-3013-3 (PMC6050736; doi:10.1186/s13071-018-3013-3)

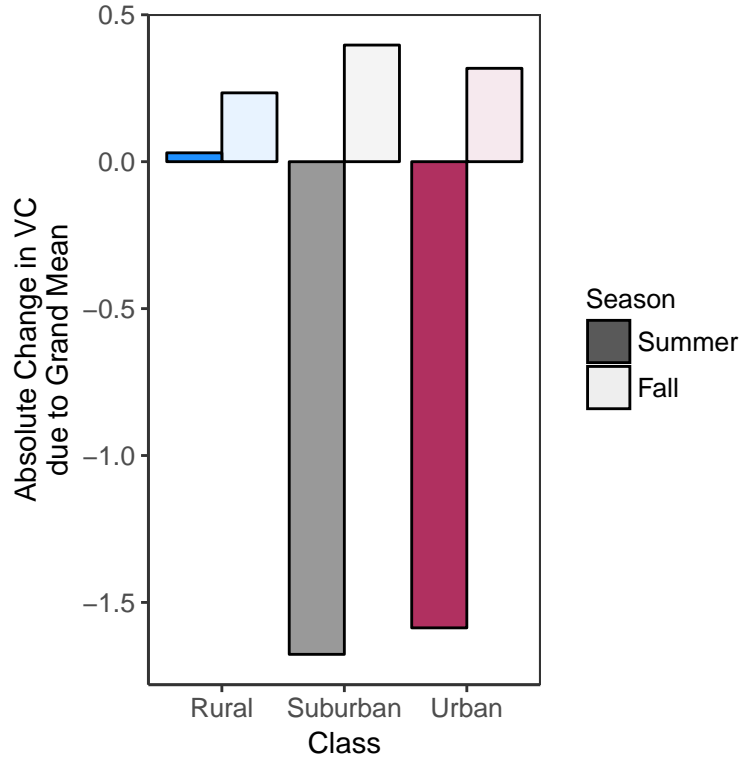

Supplement: Supplementary file 2 — Figure S1. Bias in VC due to not accounting for site-level carry-over effects across land class and season. (PDF 7 kb) [file 13071_2018_3013_MOESM2_ESM.pdf]
